# Honeybee Colony Growth Period Recognition Based on Multivariate Temperature Feature Extraction and Machine Learning

**DOI:** 10.3390/s25133916

**Published:** 2025-06-23

**Authors:** Chuanqi Lu, Lin Li, Denghua Li, Qiuying Huang, Wei Hong

**Affiliations:** 1 College of Engineering, Huazhong Agricultural University, Wuhan 430070, China; luchuanqi1987@buaa.edu.cn (C.L.); lilin@stu.bucea.edu.cn (L.L.); 2 Agricultural Information Institute of Chinese Academy of Agricultural Sciences/Key Laboratory of Agricultural Monitoring and Early Warning Technology, Ministry of Agriculture and Rural Affairs, Beijing 100081, China; 3 College of Plant Science & Technology, Huazhong Agricultural University, Wuhan 430070, China; qyhuang2006@mail.hzau.edu.cn; 4 MOE Key Laboratory of Fundamental Physical Quantities Measurement & Hubei Key Laboratory of Gravitation and Quantum Physics, PGMF and School of Physics, Huazhong University of Science and Technology, Wuhan 430074, China

**Keywords:** temperature monitoring, time domain characteristic, machine learning, growth period recognition, honeybee colonies

## Abstract

Identifying the growth period of bee colonies can guide beekeepers to make better decisions and promote the development of bee colonies. Unlike traditional manual experience-based recognition, this paper proposes a new approach, which combines multivariate temperature feature extraction and machine learning to intelligently recognize the growth period of bee colonies. Firstly, the year-round temperature data from 38 hives in Tai’an and Guilin was collected. Then, the 17 time domain characteristic indices were extracted from this dataset. To acquire the most sensitive features, the impact of different time scales on temperature feature extraction was analyzed. Subsequently, principal component analysis (PCA) was employed to reduce the dimensionality of the original feature vectors, thereby decreasing computational load and enhancing feature sensitivity. Finally, six machine learning algorithms, including both supervised and unsupervised learning, were utilized to identify the growth period of bee colonies. The results demonstrate that the proposed features can effectively characterize the growth period of bee colonies, and the BP method performs best in predicting growth period categories, with an MAE of only 1.45%. Moreover, the identification results of different regions also prove the practicability of the proposed method.

## 1. Introduction

Honeybees, as one of the most important pollinators, play a pivotal role in safeguarding wildlife species diversity and maintaining the ecological equilibrium of our planet [1]. However, the health of bee colonies is facing increasing challenges. Since 2006, approximately 30% of bee colonies in the United States have been dying annually, with winter colonies consistently collapsing over recent decades [2,3,4,5]. This phenomenon not only significantly impacts bee colony productivity but also holds immense ecological implications. Therefore, it is crucial to monitor the status of bee colonies [6]. Over the past century, scholars have employed diverse quantitative methodologies to assess an array of characteristics, such as temperature [7,8,9,10,11,12], humidity [13,14], weight [15], sound [16,17], vibration [18], and CO_2_ [19,20], and attempted to establish a relationship between the detection characteristics and bee colony activity. These efforts lay down a theoretical foundation for continuous monitoring of bee colonies [21]. Numerous past studies have shown that temperature has the greatest impact on the life activities of bees and changes in environmental temperature directly affect group activity, colony reproduction, and the population of bees [22,23]. Hence, it is imperative to investigate the temperature fluctuations within bee colonies to achieve precision beekeeping [24,25].

Studying the distribution, variation, and regulation mechanisms of colony temperature has become a hot topic in the field of bee colonies. As early as 1907, Gates et al. began to monitor temperature changes in bee colonies to guide beekeeping practices [7]. Jones et al. attempted to study the relationship between temperature and colony activities using temperature data, but only one temperature sensor was used, and the number of days and colonies monitored was relatively small [8]. With the rapid development of sensors and modern information technology, the monitoring duration and number of bee colonies have increased significantly. Kviesis et al. used a real-time colony temperature monitoring system introduced in Ref. [10] to continuously monitor the temperature within 10 hives for three years [11]. These studies have shown that the temperature stability in beehives can reflect the adaptability of bee colonies to the environment and their health status [12]. For instance, the process of the waggle dance and centralized decision-making leads to a significant increase in the temperature in the beehive, so temperature changes can be used as an indicator for the occurrence of typical events, such as bee splitting [9,26]. Although many studies have achieved long-term monitoring of colony temperature successfully, most of the current research has focused on intuitive changes in temperature and the connection between observable temperature and colony behavior, and the relationship between the internal temperature characteristics and growth periods of bee colonies has rarely been studied. In fact, bees exhibit varying behavioral patterns and face different challenges during different growth periods. For example, during the spring propagation period, temperature fluctuations and large day–night temperature differences can lead to heat loss when the hive is frequently opened, making it difficult for bees to maintain the internal hive temperature. In the autumn decline period, attention must be paid to nurturing appropriately aged winter bees and ensuring sufficient winter feed. This demonstrates that adopting different beekeeping strategies at different bees’ growth periods is beneficial for maintaining colony health. Therefore, the main goal of this study is to accurately identify the growth period of bee colonies based on the internal temperature characteristics, thereby guiding beekeepers in implementing scientific strategies for precision beekeeping.

The remainder of this paper is organized as follows. Section 2 provides the description of monitoring experiments and the method for identifying growth periods. In Section 3, the detailed results are presented. Some discussion is given in Section 4. Conclusions are drawn in Section 5.

## 2. Materials and Methods

### 2.1. Temperature Monitoring Experiments Based on Smart Beehives

This study employs a proprietary intelligent beehive monitoring system based on IoT (Internet of Things) technology [6,13]. The monitoring system employs wireless transmission technology to send data from smart hives to a remote server every hour, automatically storing it in the cloud server. It includes five sensors—temperature, humidity, nest entrance and exit count, sound, and weight—enabling it to capture diverse characteristics within the beehives.

From September 2019 to May 2022, experimental sites were established in various locations, including Tai’an, Shandong Province (east longitude 116°20′–117°59′, north latitude 35°38′–36°28′), and Guilin, Guangxi Province (east longitude 109°36′–111°29′, north latitude 24°15′–26°23′). Continuous monitoring experiments were conducted on colonies of Italian honeybees that were sourced from Tai’an and Guilin. The bee colonies selected for the experiment were procured from local beekeepers who used conventional beehives. Before any beehive replacements or relocations, a meticulous evaluation of the colonies’ health status was conducted to preclude disturbances stemming from diseases and pests.

**Figure 1:**
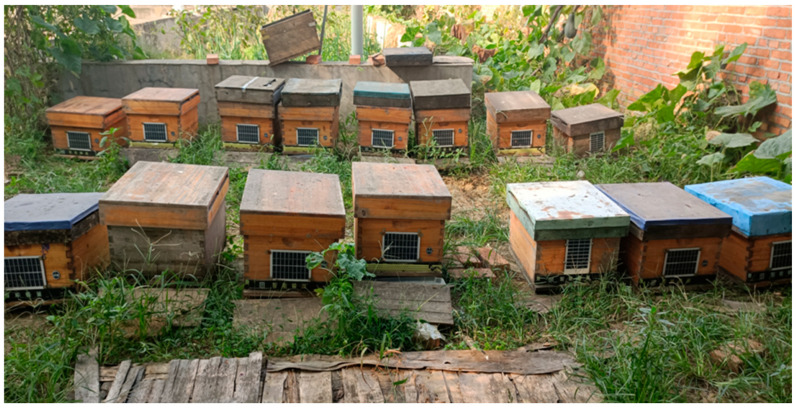
Actual deployment diagram of the smart beehives in Tai’an.

The smart beehives were used to monitor the activities of honeybees. Beehives in Tai’an and Guilin were positioned in adjacent areas to minimize potential experimental interference resulting from location disparities, as depicted in Figure 1. To ensure the stability of the experiment, preliminary experiments were conducted.

**Figure 2:**
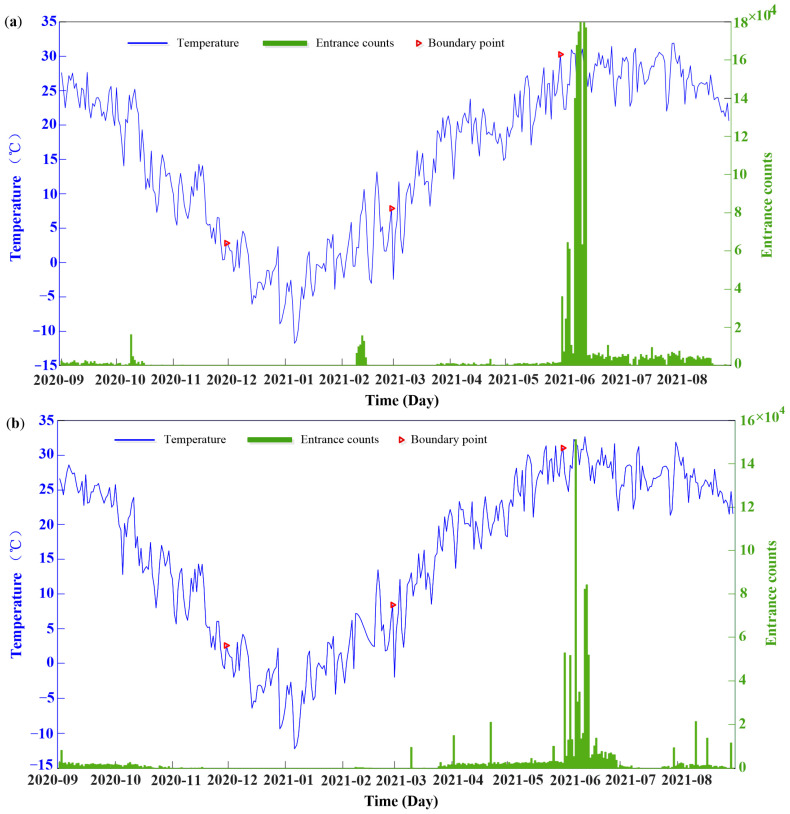
The daily monitoring results of (**a**) hive No. 7 and (**b**) hive No. 14.

Thirty-eight colonies with complete year-round monitoring data were selected. Among these bee colonies, 33 were from Tai’an and labeled as 1–33, while the remaining 5 were from Guilin and labeled as 34–38. Data were collected at hourly intervals in Beijing Time, with each data record consisting of left temperature, right temperature, and nest entry–exit counts. These data were stored in tabular form within a database. The temperature sensor is the DHT12 (Aosong Electronics, Guangzhou, China), with a measurement range of −20 °C to 60 °C and an accuracy of ±0.5 °C. The temperature referred to in this paper is the average value of the internal left and right temperatures of the hive. To visually represent the temporal variations in bee colony temperatures throughout the year, beehives No. 7 and No. 14 from Tai’an were randomly chosen for analysis, and their temperature fluctuations are illustrated in Figure 2. The population of bee colonies of two hives depicted in Figure 2 is different, but the temperature trend of the two colonies remains consistent overall. By combining temperature and entrance counts, we can roughly divide the year into 4 growing periods [6,27]. This finding is consistent with actual observations of bees in Tai’an, which proves the reliability of our monitoring experiments.

### 2.2. Temperature Feature Extraction

Currently, most studies focus on a certain temperature, such as 10 °C, associated with bees going out, and lack in-depth exploration of temperature data. Time domain analysis is a well-established signal processing method that enables us to uncover the underlying patterns within data through statistical analysis. The key to time domain analysis is to obtain its time domain indices, which are usually categorized into dimensional and dimensionless indices based on the presence of physical units. Dimensional indicators include maximum value, minimum value, peak-to-peak value, variance, mean, and root mean square, among others. Dimensional indices are straightforward but less stable in data analysis, presenting challenges in practical applications. Unlike dimensional indices, dimensionless indices, obtained by taking the ratio of two-dimensional indices with the same units, are often used to characterize both sensitivity to waveform changes and stability. Among the dimensionless indices, waveform factors, clearance factors, impulsive factors, crest factors, and skewness and kurtosis indices are widely used in other fields. By combining these commonly used dimensional and dimensionless indices, comprehensive and multi-perspective information gathering can be achieved. This approach has been widely used for extracting features in the field of pattern recognition [28,29]. Therefore, a total of 17 time domain features, including 11 dimensional indices and 6 dimensionless indices, were utilized in this paper. A more detailed explanation of these indices can be seen in our previous study [25]. To explore the impact of different time scales on temperature feature extraction, daily data analysis and hourly data analysis were adopted in this paper.

### 2.3. Feature Dimension Reduction Based on PCA

In the previous studies [6,13], it has been proved that there may be a strong correlation between the 17 indicators, such as the root mean square value and the square root amplitude. The correlation coefficients between 17 time domain indicators are calculated. Based on the calculations, it can be found that there is a strong correlation between some indicators, so it is necessary to optimize the characteristics to improve the identification accuracy and reduce computational burden. As one of the most effective dimensionality reduction methods, PCA has been used successfully in the pattern recognition field. Hence, PCA is employed in this paper to select the most sensitive features. A detailed explanation of the PCA method can be found in ref. [28]. In the PCA method, the important step is to determine the number of principal components. Generally speaking, satisfactory results can be obtained when the cumulative contribution rate of selected principal components exceeds 85% [30]. Thus, the threshold of the cumulative contribution rate is set to 0.95 in this paper.

### 2.4. Growth Period Identification Based on Machine Learning

#### 2.4.1. Unsupervised Learning Approach

Unsupervised learning can analyze unlabeled training samples to discover the structures and patterns within the training dataset. Clustering analysis, as a typical unsupervised learning method, has been widely used in the field of pattern recognition. Clustering analysis groups objects with similar features without the need for labeled training samples, making it particularly suitable for small-sample classification. Clustering is often based on distance partitioning, where an initial partition is created, and then an iterative relocation method is used to reposition individual samples until certain conditions are met. In this section, three kinds of clustering methods, including unweighted pair group average (UPGA), K-means clustering, and Fuzzy C-Means (FCM) clustering, are employed to identify the growth periods of bee colonies.

#### 2.4.2. Supervised Learning Approach

Classification is the process of separating different data based on predefined labels, which involves training a classifier on a labeled dataset to obtain good performance. Once trained, the classifier can be used to predict labels for unknown data. This method belongs to supervised learning, and common classifiers include Support Vector Machines (SVM) and Backpropagation Neural Networks (BPNNs), among other algorithms. In this paper, we mainly adopt the FCM algorithm based on TS fuzzy inference (TS-FCM), SVMs, and the BP algorithm to classify the growth period of bee colonies with supervised learning. In supervised learning models, the performance is closely related to the division of the training and test sets, as well as the selection of hyperparameters. In this study, the data was randomly split into training and test sets in a 3:1 ratio. The selection of hyperparameters was primarily determined using empirical methods, k-fold cross-validation, and grid search.

## 3. Results

### 3.1. Temperature Feature Analysis

**Figure 3:**
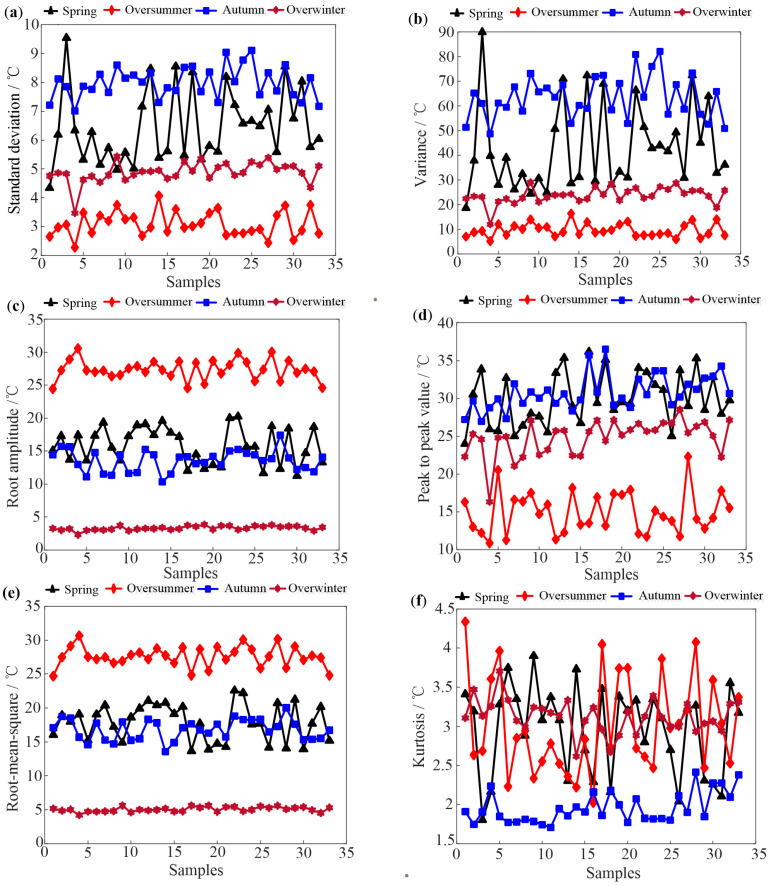
Some typical dimensional time domain indicators of the 33 hives. (**a**) standard deviation (**b**) variance (**c**) root amplitude (**d**) peak to peak value (**e**) root-mean-square (**f**) kurtosis.

**Figure 4:**
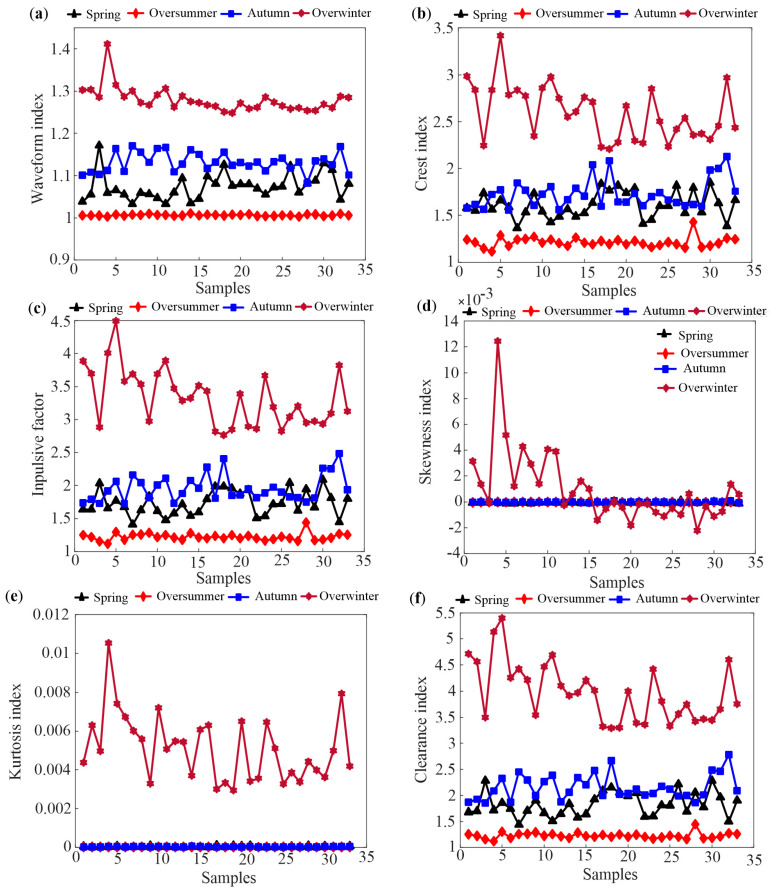
Some typical dimensionless time domain indicators of the 33 hives. (**a**) waveform index (**b**) crest index (**c**) impulsive factor (**d**) skewness index (**e**) kurtosis index (**f**) clearance index.

Based on the feature extraction method in Section 2.2, the time domain indices of 33 hives in Tai’an at different growth stages are obtained. Figure 3 and Figure 4 depict some typical dimensional and dimensionless indicators of the four growth periods based on daily data analysis, respectively.

As shown in Figure 3, it can be observed that the oversummering and overwintering periods are easily identified, while distinguishing between the spring prosperity period and autumn decline period is challenging due to similar temperature change patterns. In Figure 4, skewness and kurtosis indicators of four growth periods are completely confused. Comparing Figure 3 and Figure 4, the waveform indicators perform the best in distinguishing the four growth periods among all the indicators. This is because waveform indicators represent the degree of deviation in temperature data, indicating the stability of the waveform and the presence of significant fluctuations. Nevertheless, the results shown in Figure 3 and Figure 4 suggest that it is difficult to use a single indicator that can distinguish all four growth periods.

**Figure 5:**
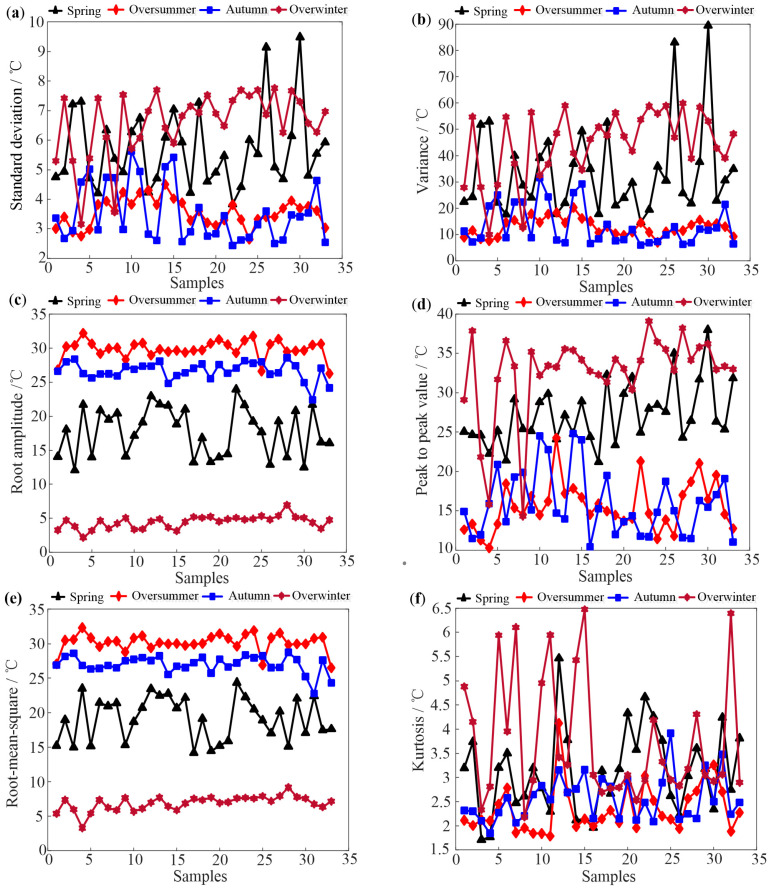
Dimensional time domain indicators of hourly temperature data. (**a**) standard deviation (**b**) variance (**c**) root amplitude (**d**) peak to peak value (**e**) root-mean-square (**f**) kurtosis.

**Figure 6:**
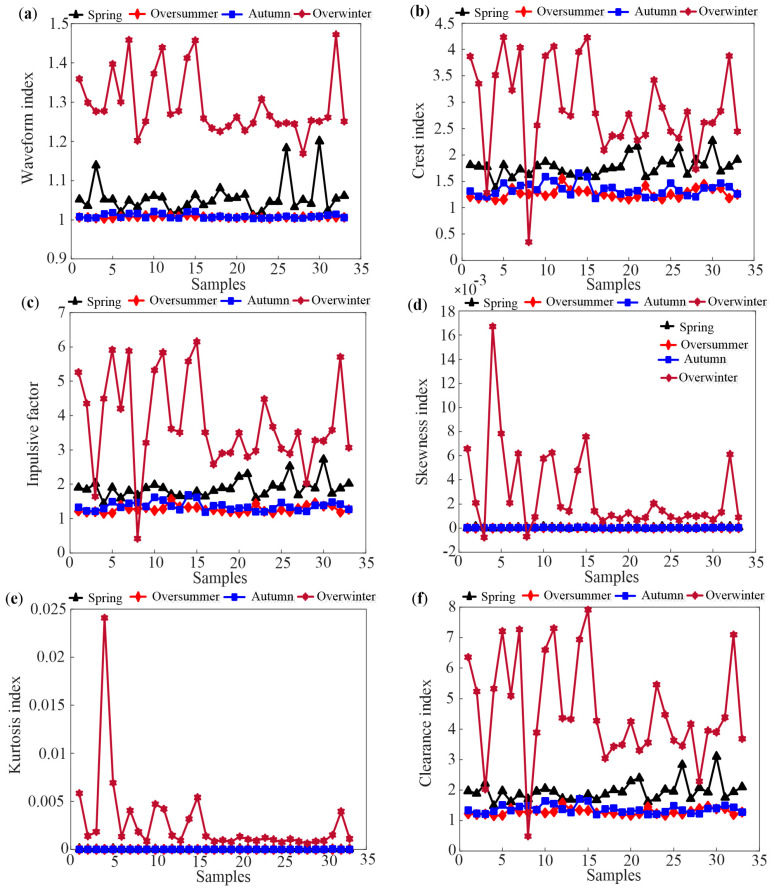
Dimensionless time domain indicators of hourly temperature data. (**a**) waveform index (**b**) crest index (**c**) impulsive factor (**d**) skewness index (**e**) kurtosis index (**f**) clearance index.

In order to explore the influence of different time scales on temperature feature extraction, hourly data analysis is conducted using typical weekly temperature data of different growth periods. Specifically, the weeks of 10–16 September, 9–15 January, 24–30 April, and 4–10 August are chosen to represent the autumn decline, overwintering, spring propagation, and oversummering periods, respectively. Instantaneous temperatures of bee colonies are recorded every hour during these periods. The experimental subjects and samples remain consistent with those in daily data analysis, and the difference is that features are extracted from 168 h of temperature data under an hourly scale. This adjustment aims to capture local temperature variations within each day, providing a more nuanced understanding of the temperature dynamics throughout the week and potentially improving the ability to differentiate between the four growth periods. Figure 5 and Figure 6 depict dimensional and dimensionless indicators of the four growth periods, respectively, under hourly time scales.

Comparing Figure 5 with Figure 3, it is evident that these indicators extracted from hourly data fail to accurately distinguish between the oversummering and autumn decline periods. This discrepancy arises because the computation of time domain indicators for hourly temperature significantly relies on the selection of representative weeks. In this case, the early August and early September weeks chosen as representative for the summering and autumn decline periods exhibit nearly identical daily temperature variations, leading to an inability to clearly differentiate between the two periods.

Compared with Figure 4, the same results can be found in Figure 6. It is obvious that the distinguishing ability of all dimensionless indexes decreased significantly. Moreover, the indicators of the overwintering period fluctuate seriously, and the possible reason is the sensitivity of dimensionless indicators to waveform changes, especially considering the significant temperature variations within a day during the overwintering period.

Upon comparing the differentiation effects of two different time scales, it is observed that the features extracted from daily average temperature are superior to those extracted from hourly temperature when distinguishing growth periods. Considering this, the time domain features of the daily average temperature for bee colonies are selected in this paper.

### 3.2. PCA Analysis

As the number of beehives from Tai’an is 33 and each feature vector consists of 17 time domain feature indices, 132 sets of feature vectors (132 × 17) are obtained. Subsequently, PCA is applied to reduce the dimensionality of the original feature vectors. After calculating the contribution rates of each principal component, we found that the cumulative contribution rate of the first three principal components had reached 99.67%. Hence, the first three principal components obtained from PCA are used to replace the original 17-dimensional feature vector.

### 3.3. Growth Period Identification Results

#### 3.3.1. Unsupervised Learning Results

**Figure 7:**
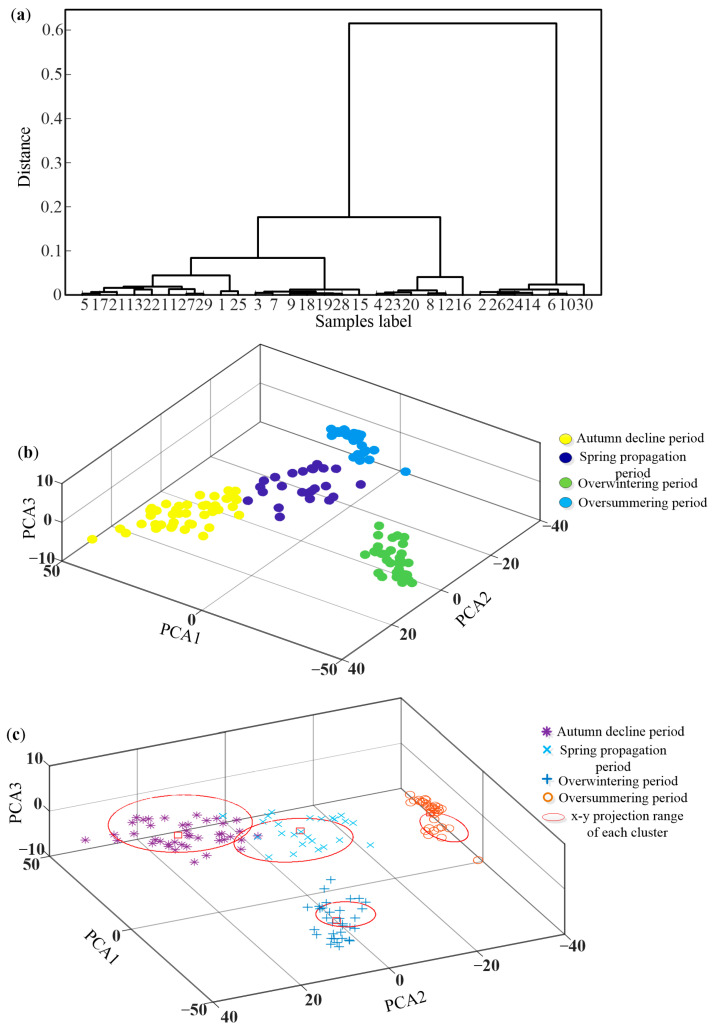
The clustering results of (**a**) UPGA, (**b**) K-means, and (**c**) FCM.

Three clustering methods depicted in Section 2.4 are used to classify the 132 samples obtained from PCA, and the clustering results are shown in Figure 7.

As illustrated in Figure 7, both oversummering and overwintering periods can be accurately identified by the three methods, and a different degree of aliasing occurs in both spring propagation and autumn decline periods. The possible reason is that the daily average temperature changes in spring and autumn are similar. Comparing these three methods, the recognition accuracy of all methods is above 90%, with the highest being the UPGA method, reaching 93.12%. The detailed calculation can be found in the Supplementary Materials.

#### 3.3.2. Supervised Learning Results

**Figure 8:**
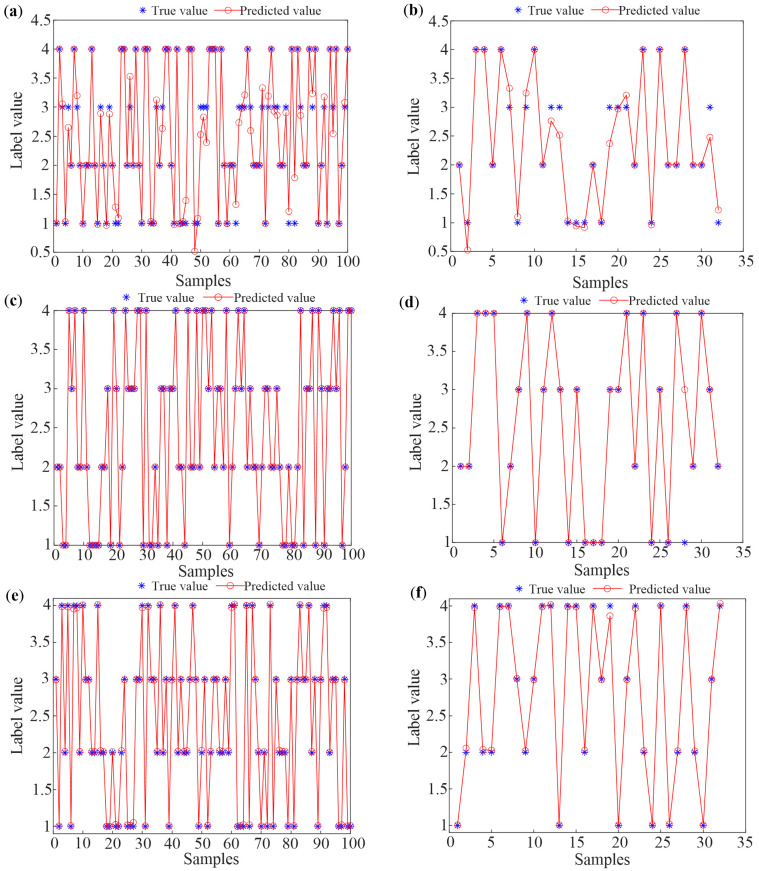
The classification results of training samples based on (**a**) TS-FCM, (**c**) SVM, and (**e**) BP and the results of testing samples using (**b**) TS-FCM, (**d**) SVM, and (**f**) BP.

First, the samples, obtained from four growth periods of 33 colonies in Tai’an, are labeled 1 (spring propagation period), 2 (oversummering period), 3 (autumn decline period), and 4 (overwintering period). In order to enhance the generalization ability of the algorithms, the dataset is randomly disrupted. The first 100 samples are chosen as the training set, and the last 32 samples are the testing set. Subsequently, three methods are utilized to classify these training samples and testing samples, respectively, and the classification results are given in Figure 8.

Figure 8a,b indicate the identification results of the training samples and testing samples based on the TS-FCM method. In the TS-FCM method, the number of clustering centers is four, the segmentation matrix index is two, the maximum number of iterations is 150, and the error of iteration stop is 1 × 10^−5^. An adaptive neural fuzzy system is applied to the fuzzy clustering system, training fine tuning to evaluate the accuracy and effectiveness of the systems. In Figure 8a,b, it can be observed that the error between the output class and the actual class is large when using the TS-FCM method. Considering the uncertainty of the method, the method is repeated 10 times. At this time, the highest recognition accuracy is 90.625%.

Figure 8c,d show the classified results of the training samples and the testing samples when using the multi-classification SVM method. In this paper, the kernel function is set to the Gaussian kernel, which has shown excellent performance in pattern recognition [31]. To achieve better performance, grid search and five-fold cross-validation were used to determine the two parameters of the Gaussian kernel function, namely, the penalty coefficient and bandwidth. The optimized parameter values are 3 and 0.05, respectively. As indicated in Figure 8c,d, all training samples can be identified accurately, and the method also exhibits excellent performance for the testing samples, with only one sample incorrectly classifying the spring prosperity period as the autumn decline period. The overall accuracy for the testing samples is 96.875%.

The identification results of the BP algorithm are depicted in Figure 8e,f. In the BP method of this study [32], the Trainlm training function and Tansig transfer function are adopted, the number of the maximum training steps is 1000, the learning rate is 0.01, the goal of training error is 0.0001, and the hidden nodes are 10. As can be seen in Figure 8e,f, the difference between the predicted values and the true values is relatively small for both training samples and testing samples; this shows that the BP algorithm can identify the growth periods of bee colonies accurately.

**Figure 9:**
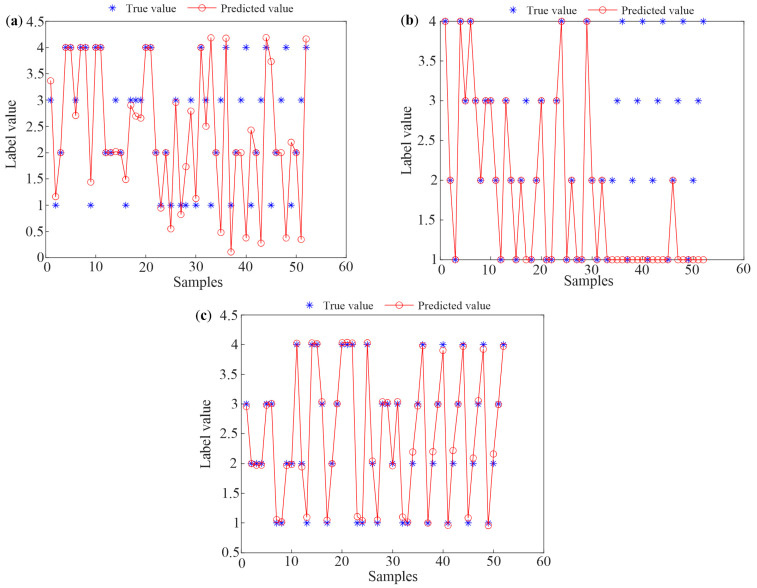
The identification results of mixture testing samples based on (**a**) TS-FCM, (**b**) SVM, and (**c**) BP.

To verify the applicability of the proposed method, twenty temperature datasets, obtained from Guilin’s five bee colonies, are added to the test set considering the influence of different regions. The original 17-dimensional time domain characteristics of the temperature data of Guilin’s bee colonies are extracted using the same time domain feature extraction method. Then, PCA is used to reduce the dimensionality of the original eigenvector, and 20 testing samples are obtained. Figure 9 illustrates the recognition results of the three methods after adding the testing samples from Guilin.

In Figure 9, one can see that the recognition accuracy of the TS-FCM and SVM methods decreases significantly and is only 63.46% and 71.15%. In Figure 9b, it can be found that the newly added Guilin testing samples can hardly be accurately identified. Comparing Figure 9c with Figure 8f, the deviation between the predicted values and the real values increases, but the BP algorithm can still identify the testing samples accurately.

To further quantitatively evaluate the recognition performance of the three algorithms, three indicators, including the mean squared error (MSE), mean absolute error (MAE), and mean relative error (MRE), are used in this paper. Table 1 shows the calculated values of the errors of the three methods. In Table 1, we can find that most of the indexes of the BP method are the best. Meanwhile, all errors have increased significantly after adding the testing samples from Guilin, which indicates that the newly added test sample has a large impact on the trained prediction model.

## 4. Discussion

This study demonstrates an improvement in the identification of honeybee (Apis mellifera) colony growth periods through advanced feature extraction techniques and machine learning algorithms. Our results (Figure 3, Figure 4, Figure 5 and Figure 6) show that daily mean temperature data demonstrates significantly superior performance in growth periods classification compared to hourly measurements, consistent with previous findings [13,24]. However, as noted in Ref. [9], high-temporal-resolution data provide distinct advantages for analyzing fine-scale behavioral patterns. This seeming contradiction may be explained by the differential temporal alignment with biological rhythms: daily averages better correspond to developmental timescales, while hourly resolution captures transient behaviors, such as swarming events (as demonstrated in prior swarming studies). Furthermore, hourly data are contaminated by substantial noise from environmental fluctuations (e.g., sudden weather changes), whereas daily averaging effectively attenuates these stochastic disturbances through temporal integration, thereby more reliably reflecting colony-level physiological states. Unsupervised learning analyses further substantiate these findings, showing that temporal features extracted from daily temperature data effectively discriminate growth periods. Moreover, the validation results from supplementary datasets (acoustic intensity/count data) shown in the Supplementary Materials further confirm the dominant role of temperature dynamics in growth stage identification.

The assessment of the honeybee colony growth period currently relies primarily on the experiential judgment of beekeepers. We have not identified any studies focusing on intelligent and precise identification in this area, making it difficult to compare our results with other methods. Nonetheless, our achievements in the intelligent identification of the honeybee colony growth period are satisfactory, particularly in terms of promoting precision beekeeping.

The limitations of this work are as follows. Firstly, the bee colonies used in this study are mainly Italian bees from Tai’an, with a small portion from Guilin, which may limit the generalizability of the results. Future research needs to consider the impact of differences in bee varieties and climate changes outside these regions [12]. Additionally, the experience and management practices of beekeeping experts in each area may also influence data labeling. However, with the gradual adoption of smart beehives, standardized collection processes will effectively reduce these potential errors. Furthermore, this study only used temperature monitoring data to extract characteristic values, and although satisfactory identification results were achieved, the growth phase of bee colonies can also be reflected in changes in humidity, sound, frequency of bees entering and exiting the hive, and comb weight [20]. Future research should focus on integrating multi-source information to guide precision beekeeping.

In summary, the dynamic characteristics of temperature have been proven to effectively distinguish the growth periods of bee colonies. For future research, we can further explore the relationship between temperature dynamics and bee colony behavior. Specifically, we aim to gain a deeper understanding of bee behavior patterns under different temperature conditions, including changes in foraging, nesting, and colony activity. By systematically analyzing the specific effects of temperature fluctuations on bee behavior and identifying key temperature thresholds and their characteristics, we can provide more targeted guidance for scientific beekeeping practices.

## 5. Conclusions

In this study, an effective growth period recognition approach has been proposed. Unlike the traditional temperature monitoring analysis reported in many other studies, the main objective of this study is the development of methods that can reliably identify the growth periods of bee colonies. Because bee colonies have different growth and behavior patterns in different growth stages, the proposed method is very important in precision beekeeping applications, as it enables accurate determination of the critical growth period and properly plans beekeeping measures in advance. According to the aforementioned illustration, the proposed scheme involves multivariate time domain feature extraction, a PCA for feature dimension reduction, and an identification of growth periods based on machine learning. Based on long-term monitoring experiments of 38 bee colonies and comparative analysis of different machine learning algorithms, we can conclude the following.

The time domain features extracted from the daily mean temperature series are superior to those extracted from the hourly temperature series when characterizing different colony growth periods, and a single time domain feature could not effectively distinguish the spring blooming period from the autumn declining period.

Comparing common supervised and unsupervised machine learning methods, it can be found that the BP algorithm can identify the growth period of bee colonies with 100% accuracy, even considering the differences in the geographical locations of bee colonies.

## Figures and Tables

**Table 1:** Error index values of the three supervised methods.

Performance Index	Testing Samples of Tai’an			Testing Samples of Tai’an and Guilin		
	TS-FCM	SVM	BP	TS-FCM	SVM	BP
MSE	0.0359	0.0303	0.0004	0.4958	0.4803	0.0031
MAE	0.0951	0.0152	0.0145	0.2563	0.2039	0.0405
MRE	0.0546	0.0051	0.0070	0.4033	0.2039	0.0242

## Data Availability

The original contributions presented in this study are included in the article/Supplementary Material. Further inquiries can be directed to the corresponding authors.

## Supplementary Materials

The following supporting information can be downloaded at https://www.mdpi.com/article/10.3390/s25133916/s1, Figure S1: Correlation coefficient matrix; Figure S2: The contribution rate of each principal component; Figure S3: The identification results of multi-source data based on TS-FCM; Figure S4: The identification results of multi-source data based on GS-SVM; Figure S5: The identification results of multi-source data based on GA-SVM; Figure S6: The identification results of multi-source data based on BP; Table S1: Recognition results of the three unsupervised clustering methods; Table S2: Eleven dimensional indices of some honeybee colonies; Table S3: Six dimensionless indices of some honeybee colonies.
