# Supplementary material for: Honeybee Colony Growth Period Recognition Based on Multivariate Temperature Feature Extraction and Machine Learning
Source: Sensors (Basel). 2025 Jun 23;25(13):3916. doi: 10.3390/s25133916 (PMC12251692; doi:10.3390/s25133916)
Supplement: Supplementary file 1 [file sensors-25-03916-s001.zip › sensors-3665623-supplementary.pdf]

(**1**) The clustering results of 132 samples using the UPGA method are as follows:

Label 1: 3 7 9 11 15 19 23 27 31 35 39 43 47 49 51 55 59 61 63 67 69 71 75 79 83 85 87 91 95 99 101 103 107 111 113 115 117 119 121 123 127 131

Label 2: 1 5 13 17 21 25 29 33 37 41 45 53 57 65 73 77 81 89 93 97 105 109 125 129

Label 3: 4 8 12 16 20 24 28 32 36 40 44 48 52 56 60 64 68 72 76 80 84 88 92 96 100 104 108 112 116 120 124 128 132

Label 4: 2 6 10 14 18 22 26 30 34 38 42 46 50 54 58 62 66 70 74 78 82 86 90 94 98 102 106 110 114 118 122 126 130

Based on the clustering results, we can find that nine samples marked in red belonging to Label 2 are classified into Label 1, so the overall accuracy is 123/132=93.2%.

(**2**) The recognition results of three unsupervised clustering methods are given in Table S1.

Table S1. Recognition results of the three unsupervised clustering methods.

	UPGA				K-means				FCM			
True label	Predicted label				Predicted label				Predicted label			
	1	2	3	4	1	2	3	4	1	2	3	4
1	24	9			23	10			24	9		
2		33			1	32			1	32		
3			33				33				33	
4				33				33				33

Similarly, the accuracy of K-means and FCM is 91.7% (121/132) and 92.4% (122/132).

(**3**) The correlation coefficient matrix of 17 time domain features is shown in Figure S1.

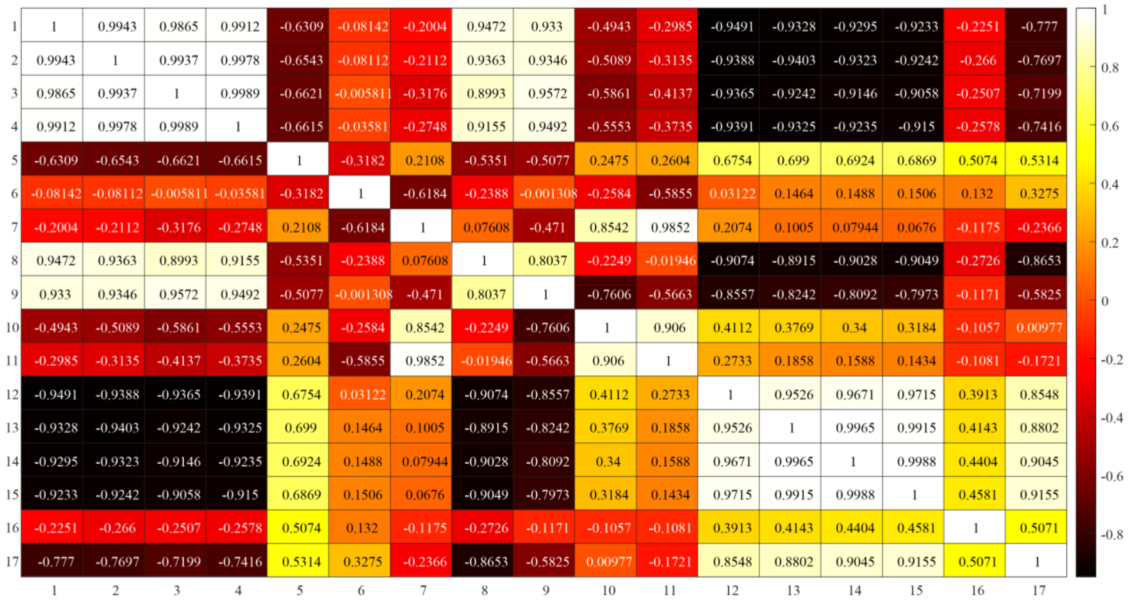

Figure S1. Correlation coefficient matrix.

As shown in Figure S1, it can be seen that there is a strong correlation between some indicators. To improve the identification accuracy of extracted features and reduce computational burden, PCA is employed in this paper. Figure S2 indicates the contribution rates of each principal component. From Figure S2, we can find that the cumulative contribution rate of the first three principal components has reached 99.67%.

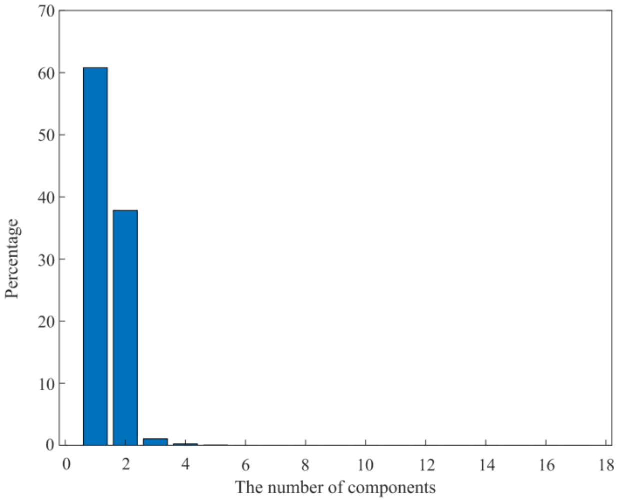

Figure S2. The contribution rate of each principal component.

(**4**) Table S2 and Table S3 show the dimensional and dimensionless indices of some honeybee colonies, respectively.

Table S2. Eleven dimensional indices of some honeybee colonies.

No.	Period	X_{mean}	X_{rms}	X_r	$\mid X_{mean} \mid$	$X_{skewness}$	$X_{kurtosis}$	$X_{variance}$	X_{max}	X_{min}	X_{vpp}	$X_{standard}$
3	Spring propagation	15.277	17.983	13.692	15.353	0.305	1.803	89.988	31.25	-2.6	33.85	9.538
7	Overwintering	-0.162	4.721	3.089	3.67	0.126	3.339	22.262	13.15	-11.8	24.95	4.745
14	Overwintering	-0.095	4.890	3.276	3.784	-0.031	3.132	23.908	13.45	-12.25	25.7	4.917
22	Oversummering	24.651	24.825	24.558	24.651	-0.529	4.408	8.642	30.45	13.5	16.95	2.956
26	Spring propagation	13.128	14.259	12.504	13.21	-0.189	3.333	30.991	25.55	-3.75	29.3	5.597
29	Autumn decline	16.045	18.264	14.673	16.119	-0.108	1.821	76.112	31.85	-1.8	33.65	8.773
34	Spring propagation	19.479	21.256	18.414	19.538	-0.246	2.308	72.391	32.6	-2.7	35.3	8.555
38	Oversummering	24.65	24.805	24.574	24.653	-0.433	3.375	7.508	30.9	15.4	15.5	2.755

Table S3. Six dimensionless indices of some honeybee colonies.

No.	Period	Waveform index	Crest index	Impulse factor	Clearance index	Skewness index	Kurtosis index
3	Spring propagation	1.171	1.738	2.035	2.282	5.24E-05	1.72E-05
7	Overwintering	1.286	2.785	3.583	4.258	0.001	0.007
14	Overwintering	1.262	2.750	3.472	4.105	-2.51E-04	0.00548
22	Oversummering	1.007	1.227	1.235	1.240	-3.46E-05	1.07E-05
26	Spring propagation	1.079	1.792	1.934	2.043	-6.53E-05	8.06E-05
29	Autumn decline	1.133	1.744	1.976	2.171	-1.76E-05	1.64E-05
34	Spring propagation	1.088	1.534	1.669	1.770	-2.56E-05	1.13E-05
38	Oversummering	1.006	1.246	1.253	1.257	-2.83E-05	8.92E-06

(5) We have incorporated additional datasets, including acoustic intensity and counts, which align closely with our original analysis objectives. Weight data was initially considered but excluded due to insufficient sample size, which would compromise statistical reliability. The expanded results are presented in Supplementary Materials as follows.

First, the samples, obtained from four growth periods of 33 colonies in Tai 'an, are labeled 1 (spring propagation period), 2

(oversummering period), 3 (autumn decline period), and 4 (overwintering period). Here, the samples consist of extracted 17-dimensional dynamic temperature features, average acoustic intensity, and mean counts. Principal component analysis (PCA) was applied to reduce the original 19-dimensional features. The results indicate that the first three principal components collectively account for over 85% of the variance, allowing the original feature vectors to be reduced to three dimensions. In order to enhance the generalization ability of the algorithms, the dataset is randomly disrupted. The first 100 samples are chosen as the training set, and the last 32 samples are the testing set. Subsequently, three methods are utilized to classify these training samples and testing samples, respectively, and the classification results are given in Figures 3-6.

As shown in Figures S3-S6, both TS-FCM and SVM exhibit significantly lower recognition accuracy across both training and testing samples compared to using temperature dynamic features alone, while the BP algorithm maintains precise identification of all growth stages. This phenomenon may be attributed to (1) BP's ability to dynamically adjust feature weights through backpropagation and (2) its hidden layers' capacity to construct nonlinear feature combinations—capabilities that other methods inherently lack. Furthermore, the observed accuracy degradation across all methods when incorporating new data may be associated with noise interference in the additional data. Future research will focus on optimizing feature extraction methods to enhance model robustness.

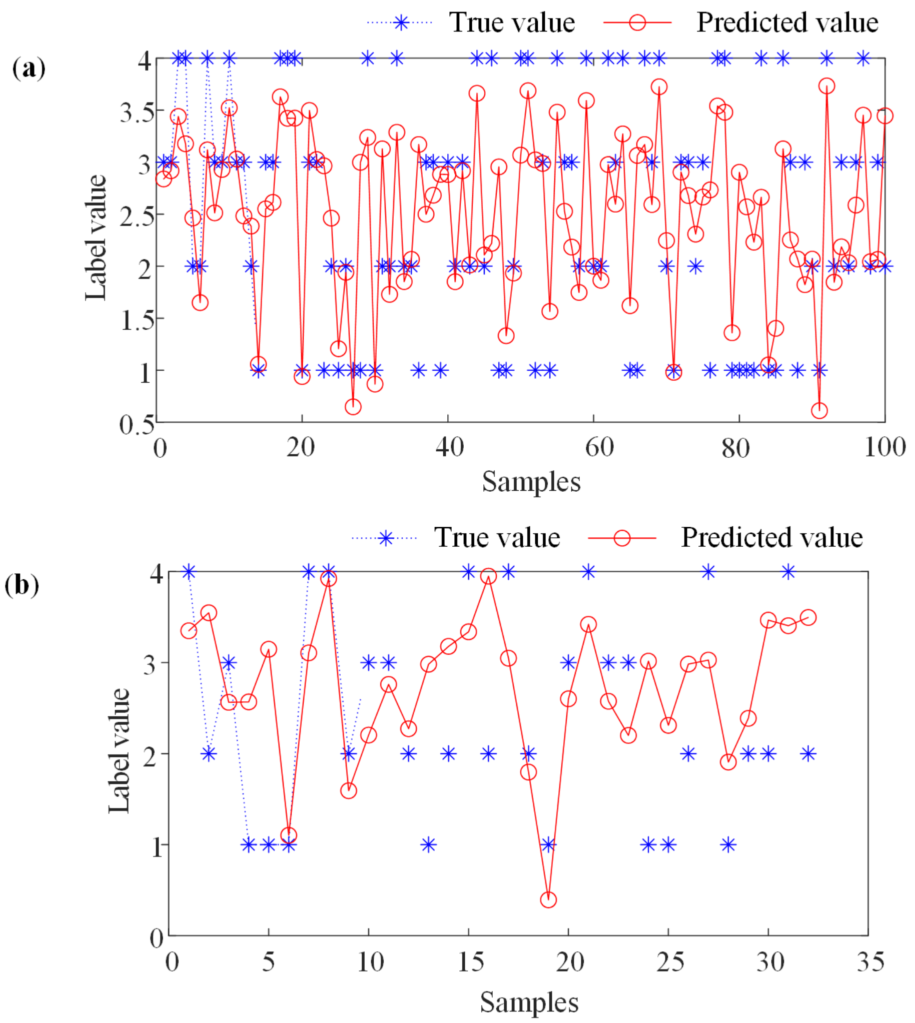

Figure S3. The identification results of multi-source data based on TS-FCM: (a) training samples and (b) testing samples.

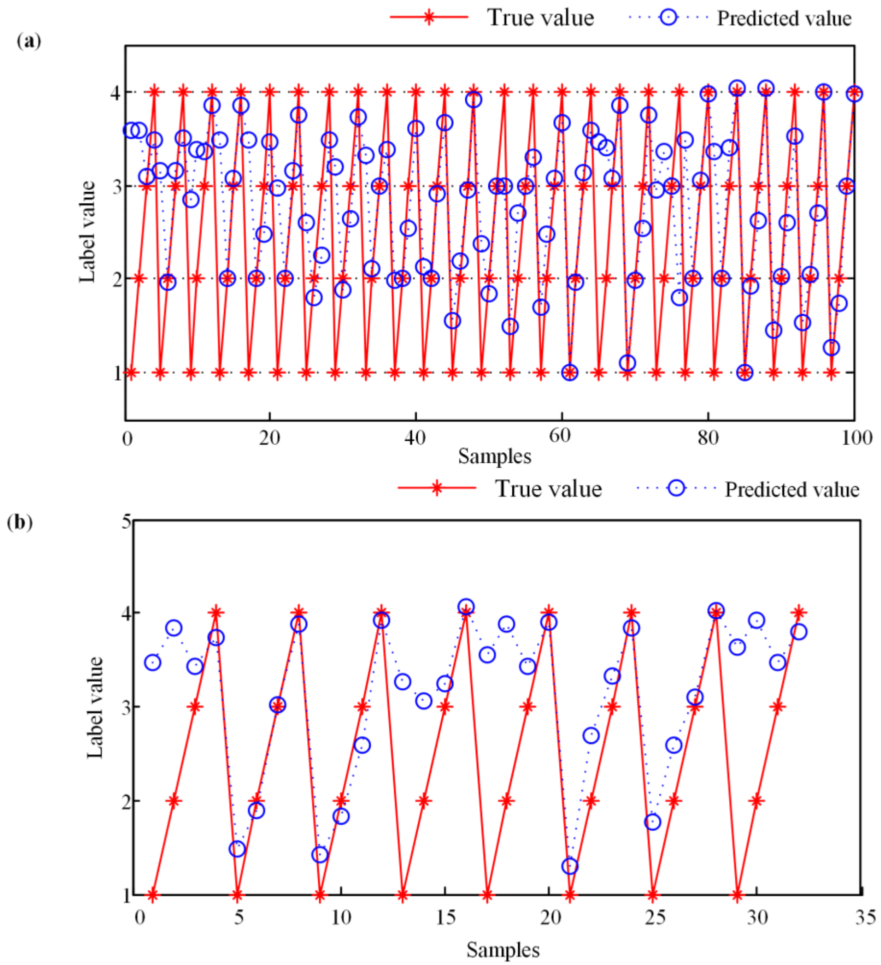

Figure S4. The identification results of multi-source data based on GS-SVM: (a) training samples and (b) testing samples.

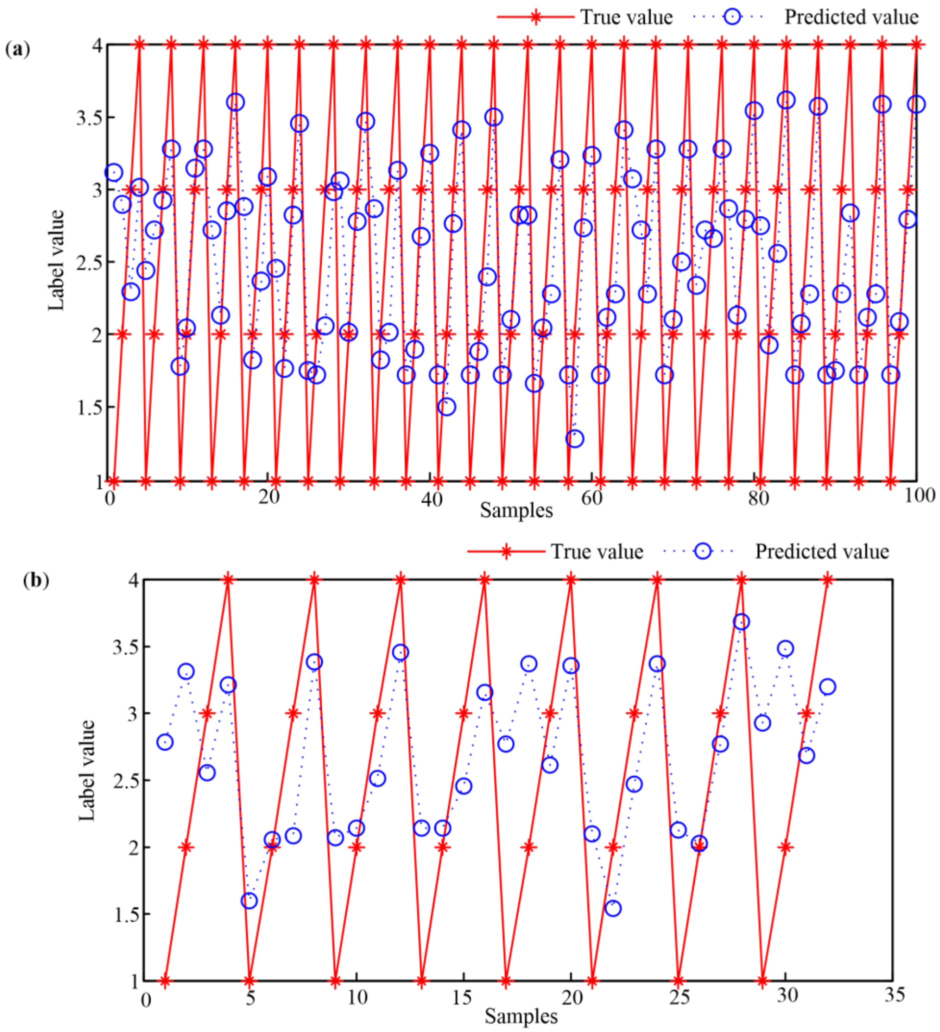

Figure S5. The identification results of multi-source data based on GA-SVM: (a) training samples and (b) testing samples.

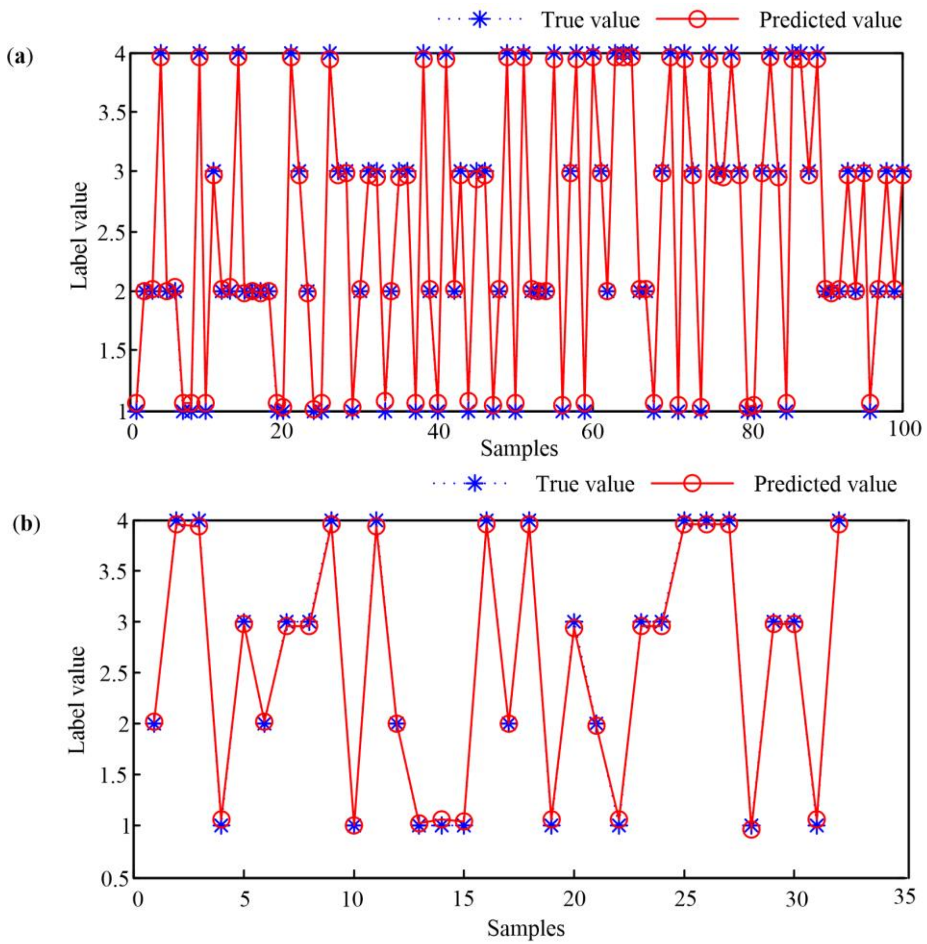

Figure S6. The identification results of multi-source data based on BP: (a) training samples and (b) testing samples.

Additionally, the dataset obtained through dimensionality reduction of multi-source data is provided as supplementary material in the Appendix.

888.2508444	-10.9963278	-144.0695848	1
967.6069885	-39.61682449	-114.8037799	2
1022.876454	-16.51559725	-113.3365411	3
852.1966419	-26.69745174	-103.1818032	4
1135.404565	-34.17469729	-115.8817306	1
2937.920094	-669.2239398	906.5103421	2
1811.350145	-36.77833689	-250.666892	3
1362.735894	-61.95154452	-151.0217099	4
1157.943919	14.28563488	-162.0551984	1
2301.984947	-126.5173842	-254.3667421	2
1663.136685	-18.7904616	-264.4652149	3
989.7031239	-1.412470525	-201.1203679	4
1067.073185	2.831798908	-188.293828	1
8544.193846	-333.9371799	-1428.277799	2
529.5780779	23.99329049	-83.91892687	3
766.9756279	-6.603140384	-153.0022349	4
934.722543	-10.88714812	-141.9558288	1
2630.814915	-332.552796	173.9142708	2
1186.223876	-72.89965554	-4.201492779	3
809.6532835	-25.07851593	-96.17436488	4
1170.475092	-50.85874913	-81.02847714	1
3324.644324	-841.4129072	1221.766932	2

1451.107784	-28.86941937	-185.5648333	3
1402.386503	-42.03474943	-212.6040319	4
1180.304922	-118.2791605	56.11436175	1
2817.694357	-553.7999625	662.9894882	2
1116.833579	-76.25002705	35.39313352	3
857.5458639	-31.30960279	-98.36337809	4
1545.33389	-73.09279889	-144.0152138	1
2794.725341	-407.4349754	306.7837759	2
1315.316137	-71.84859527	-46.41007501	3
1464.258099	-47.70656802	-216.5046668	4
854.1957633	-21.82721048	-96.98248494	1
1800.187773	-250.7677272	197.4458433	2
1113.455034	3.685783164	-151.6116372	3
1552.146934	-74.30420448	-161.0103194	4
1773.772713	-225.5808677	169.2162421	1
3902.896326	-820.7949971	1024.368356	2
1604.146064	-88.65329227	-70.43749856	3
834.8077674	-15.26295383	-129.8681662	4
1963.243002	-233.5867741	127.7789634	1
3713.572796	-709.6048118	804.5184989	2
1652.340203	-50.06298782	-174.051226	3
1423.932078	-51.12921919	-195.6606496	4
2893.614576	-305.5132173	101.5028132	1
1817.54422	-268.5146656	219.2040411	2
1315.458533	-30.06837929	-138.023243	3

1285.568869	-13.26376719	-248.2612559	4
1825.649075	-98.29388025	-89.10959635	1
4713.686598	-997.7767412	1236.943969	2
1707.42575	-41.60708155	-206.2173167	3
1307.204291	-108.243888	-21.48049354	4
2761.917345	-501.5651959	581.1251759	1
2652.491976	-198.3890253	-154.6021248	2
1142.684963	-35.31934691	-99.44856616	3
1318.858023	-77.6076699	-100.6022033	4
3519.837682	-834.877387	1197.195824	1
1332.316077	-184.5029661	145.373118	2
1419.63051	-33.50409853	-165.0598441	3
882.2275638	-12.5126485	-148.4605387	4
5234.672087	-1100.49423	1459.417073	1
3827.63221	-439.361692	112.3422457	2
934.4832231	17.52990201	-150.9892819	3
693.8279851	-0.69042366	-123.3154607	4
750.7782341	9.399954545	-137.5687352	1
858.4496813	-44.70946121	-69.30479458	2
1279.472096	-4.28017809	-178.9842752	3
1155.634586	-4.826113007	-227.6392817	4
3416.372125	-594.2204533	696.8191335	1
3598.132906	-443.2576988	176.0141719	2
1667.943413	-71.05760293	-110.9268931	3
842.8319456	3.273006085	-171.2355722	4

1087.191727	-71.81152369	-31.24839996	1
1628.642419	-101.4495142	-133.5215041	2
1382.06498	-40.19909253	-140.5269156	3
2875.875102	-669.1517841	943.1766084	4
899.4204202	4.057037033	-157.7171055	1
4931.556319	-359.1627699	-381.0535525	2
1512.106205	-24.71813002	-195.3112351	3
1273.876125	-12.00203077	-252.7276275	4
1202.224518	-36.04488996	-144.5322499	1
1911.949209	-283.7727702	246.7854492	2
1122.987069	7.629329066	-196.6232018	3
1507.781811	-13.49330857	-305.2000919	4
3005.413504	-488.3369928	544.2684178	1
2080.567167	-348.1498678	345.8447751	2
1366.209987	-36.5684636	-107.2810404	3
1546.376141	-13.41131672	-313.2355978	4
3669.955275	-756.0488698	1000.980028	1
3432.625959	-756.7429522	987.7328829	2
2507.670707	-119.5510389	-235.771655	3
1078.307259	-39.77783778	-129.8201826	4
3334.859273	-702.147453	940.7485039	1
3882.414952	-434.4304708	75.96320138	2
2235.877047	-79.18874674	-241.1199868	3
1335.244247	-10.32152217	-269.9724628	4
2494.848574	-507.90508	691.1139007	1

1887.580944	-412.3527094	555.9172274	2
2229.272018	-39.98786226	-327.6791766	3
1514.111217	-16.39682524	-296.1535561	4
896.792906	11.40546764	-166.2843811	1
1114.953624	-21.13138944	-195.6266991	2
1131.325587	15.34015777	-211.917951	3
876.6717314	4.729627932	-178.5489233	4
3810.509456	-702.6547252	828.4167605	1
2420.550115	-566.4825753	788.6303187	2
1048.825574	2.23848973	-138.7069935	3
1242.084276	-3.007589483	-251.6844649	4
2050.986727	-408.9601877	542.375445	1
7765.013759	-2412.069797	3901.480653	2
1246.772289	-62.3243092	-42.95361984	3
1236.960393	-9.864296338	-243.2752503	4
8982.56615	-934.5597658	45.87921042	1
7784.475977	-699.0796872	-311.0562953	2
2607.698365	-37.07257012	-452.5758515	3
1847.837885	-25.77836823	-365.6270854	4
1114.894466	9.242306298	-211.7793445	1
1119.629707	-23.21896808	-197.4805751	2
1189.807997	15.68531221	-224.354956	3
1165.48198	-3.349763177	-237.0007141	4
4336.942427	-929.0636082	1261.343978	1
2127.46457	-205.8035941	-13.89523814	2

1133.127772	5.32564414	-189.0174848	3
1226.619198	-18.76453942	-220.6149115	4
4202.830195	-574.1951279	376.3137114	1
2737.447932	-223.8914029	-119.6231445	2
2182.587283	-50.98937972	-311.1323535	3
1379.822552	-20.82631133	-266.2081724	4
1127.479726	4.401684239	-215.4553085	1
1213.662606	-23.23750517	-219.3857257	2
1167.959644	11.91587113	-219.2680982	3
965.046927	2.21137918	-196.5786562	4
